# Relation between body condition score and conception rate of Japanese Black cows

**DOI:** 10.5713/ab.22.0329

**Published:** 2023-05-02

**Authors:** A. Setiaji, T. Oikawa, D. Arakaki

**Affiliations:** 1 Department of Animal Science, Faculty of Animal and Agricultural Sciences, Universitas Diponegoro, Tembalang Campus, Semarang 50275, Central Java, Indonesia; 2 Faculty of Agriculture, University of the Ryukyus, Nishihara, Okinawa 903-0213, Japan

**Keywords:** Body Condition Score, Conception Rate, Heritability, Threshold Animal Model

## Abstract

**Objective:**

This study analyzes interactions of body condition score (BCS) with other factors and the effect of BCS on estimates of genetic paremeters of conception rate (CR) in Japanese Black cows.

**Methods:**

Factors affecting CR were analyzed through the linear mixed model, and genetic parameters of CR were estimated through the threshold animal model.

**Results:**

The interactions between BCS and each season and the number of artificial inseminations (AI) was significant (p<0.05), but that between BCS and parity showed no significance for CR. High CR was observed with BCS 3 in autumn (0.56±0.01) and BCS 4 in summer (0.56±0.02). The highest CR with BCS 3 (0.56±0.02) and BCS 4 (0.55 ±0.01) was observed at first AI. With BCS 5, however, the highest CR (0.55±0.08) was observed at second AI.

**Conclusion:**

The model with BCS was notably conducive to the estimation of genetic parameters because of a low deviance information criterion of heritability that, nevertheless, was slightly lower than the model without BCS.

## INTRODUCTION

In the recent decade, the price of Japanese Black calves has doubled because of the high cost of calf production in the Japanese cow-calf operating system. High production cost is primarily due to a decline in reproductive efficiency. The low efficiency in calf-producing operations is mostly influenced by the rate of artificial insemination (AI) success [1], which is controlled by numerous factors, including sperm quality [2], season [3], AI technician [4], parity [5], health, nutrition and heat-detection methods for the herd [6]. Conception rate (CR) is one of the indicators commonly used for evaluating the success of AI in cattle.

Body condition score (BCS) is a method for the management and monitoring of the amount and accumulated relative fitness of cattle [7]. Body condition of cows after calving is closely related to AI efficiency, i.e., success of first insemination, CR, and interval from first insemination to gestation [8,9]. Assuming that the success of AI is related to BCS, the magnitude and change of BCS after calving needs to be investigated to determine whether nutritional goals are achieved and to identify potential problems associated with failure of AI [10]. Cows with poor BCS may have longer time to express estrous after calving. On the other hand, cows with high BCS are usually phenotyped as obese or overweight animals requiring plural servicings for conception and as exhibiting inefficiency of the normal reproductive cycle [11,12]. The interaction between BCS, seasonal changes and feeding regimes direcly affects the reproductive performance of cows [3]. The aim of this study was to analyze the interactions between BCS with other factors and the effect of BCS on estimates of genetic paremeters of CR in Japanese Black cows.

## MATERIALS AND METHODS

### Data collection

Insemination and pedigree records of Japanese Black cows were obtained from Artificial Insemination Center of Northern Okinawa. A data set comprised 6,034 records of AI carried out on 2,114 cows by eight AI technicians. Collected between January 2015 and December 2017 from 114 farms, the records comprised 5,003 cows with and 1,031 without BCS data. The total number of animals in the pedigree was 16,334. BCS was judged at the time of insemination and scaled using 5-point system from one (1) for attenuated animals to five (5) for obese animals [13,14]. CR was coded 1 if the cow was inseminated and subsequently concieved successfully, otherwise 0. Cow was declared as conceived after two evaluations: non-return at 21 days after AI, and palpation per rectum at 35 to 40 days after AI. Approval from the committee on the care and use of animals was not sought because data used in this study were collected from field records; no field experiments were conducted.

### Categories and statistical analysis

The number of records were BCS 2 (n = 349), BCS 3 (n = 2,448), BCS 4 (n = 2,079), and BCS 5 (n = 127). Parity was coded 1 (n = 848), 2 (n = 877), 3 (n = 740), 4 (n = 536), 5 (n = 593), 6 (n = 525), 7 (n = 509), 8 (n = 449), 9 (n = 328), 10 and more (n = 629). AI was conducted in and coded “spring” (March to May; n = 1,814), “summer” (June to August; n = 1,416), “autumn” (September to November; n = 1,452) and “winter” (December to February; n = 1,352). AI was designated as first AI (n = 3,482), second AI (n = 1,370), third AI (n = 574), and fourth or subsequent AI (n = 608).

GLIMMIX procedure with contrasts of statistical analysis system 9.3 (SAS) [15] was used to analyze the effect of the interaction between BCS and parity, BCS and season, and BCS and AI number. Farm was treated as a random effect. The linear model was as follows:


$$y_{\mathrm{ijklmn}}=B_{i}+P_{j}+S_{k}+A_{l}+{\left(\mathrm{BP}\right)}_{\mathrm{ij}}+{\left(\mathrm{BS}\right)}_{\mathrm{ik}}+{\left(\mathrm{BA}\right)}_{\mathrm{il}}+c_{m}+f_{n}+e_{\mathrm{ijklmn}}$$

where ***y****_ijklmn_* is the observation of CR, ***B****_i_* the *i*th fixed effect of BCS, ***P****_j_* the *j*th fixed effect of parity, ***S****_k_* the *k*th fixed effect of season, ***A****_l_* the lth fixed effect of AI number, **(*BP*)***_ij_*, (***BS***)*_ik_*, and (***BA***)*_il_* are effects of interactions between *i*th of BCS with *j*th effect of parity, *k*th effect of season and *l*th effect of AI number, recpectively, ***c****_m_* is the random effect of cow/animal, ***f****_n_* is the random effect of farm and ***e****_ijklmn_* the random residual of ***y****_ijklmn_*.

### Estimate of genetic parameters

Genetic parameters of CR were estimated through single animal model, in matrix notation the mixed linear model was:


$$y=\mathrm{Xb}+Z_{1}a+Z_{2}\mathrm{pe}+e$$

where, ***y*** is the a vector of CR, ***b*** is a vector of fixed effect, **X** is an incidence matrix for the fixed effect, a is the vector of random genetic additive effect, ***pe*** is the vector of permanent environmental effect, ***Z****_1_* and ***Z****_2_* are incidence matrix, and e is the residuals vector.

This analysis was repeated twice; the first with BCS as fixed effect and the second without BCS to know the impact of BCS on genetic parameters estimated.

The Bayesian procedure [16] assuming that the normal distribution with density as:


$$\begin{array}{l} p(a|\sigma_{a}^{2})\sim N(O,A\sigma_{a}^{2}),\\ p(\mathrm{pe}|\sigma_{pe}^{2})\sim N(O,I\sigma_{pe}^{2})\;\text{and}\\ p(e|\sigma_{a}^{2})\sim N(O,I\sigma_{e}^{2}), \end{array}$$

where ***A*** is the numerator relationship matrix of the additive genetic relation between individuals in the pedigree; ***I*** the identity matrix; $\sigma_{a}^{2},\sigma_{\mathrm{pe}}^{2}$ and $\sigma_{e}^{2}$ the additive genetic variance, permanent environmental variance and residual variance, respectively. The variance covariance components were estimated through Bayesian procedures THRGIBBS1F90 [17], that yielded a period of data collection of 1,000,000 iterations after a burn-in period of 100,000 iterations. Values of standard error were estimated through POSTGIBBS1F90 of the Geweke diagnostic test [18]. A posteriori distribution was obtained with 4,000 samples which were taken for every 250 cycles. This estimation was repeated twice; the first with BCS and the second without BCS analysis. Geweke criteria and error of Monte Carlo chain (MCE) were obtained by calculating the variance of samples for each component divided by the number of samples to monitor the convergence.

## RESULTS

The reproductive performance of cows from 114 farms is summarized in Table 1. BCS, and parity were statistically significant (p<0.05) for CR, whereas season and AI number were not. The interaction between BCS and between season and BCS and AI number was significant, however, that between BCS and parity was not significant (Table 2). Accordingly, multiple comparisons between BCS, and season; BCS, and AI number were tested afterwards.

In terms of the interaction between BCS and the season for CR, the BCS showed no significant difference among the seasons. Nevertheless, significant seasonal differences were observed in BCS 3 and 4. A high CR was seen with BCS 3 in autumn and with BCS 4 in summer (Table 3).

The interaction between BCS and AI number is shown in Table 4. The effect of BCS, AI number and their interactions were significant differences. Significant differences of AI number were observed in BCS 3, BCS 4, and BCS 5. The highest CR with BCS 3 was observed in cows at first AI, and CR decreased as the AI number increased. With BCS 5, the highest CR was seen in cows at second AI. Also, significant differences among the BCS were observed only at the second and third AI.

In comparing estimated heritability between animals with and without BCS, the latter was slightly higher than the former (Table 5). Heritability with BCS showed a low posterior standard deviation. Values for Geweke (p-value), MCE, and deviance information criterion (DIC) for estimated heritability with BCS were lower than that without BCS.

## DISCUSSION

The mean CR of the Japanese Black cow in this study (0.52± 0.01) was slightly higher than previously reported (0.48±0.02) [19]. Compared with other breeds, CR in Japanese Black was slightly lower than the estimates of beef cows in New Zealand (0.55) [20] and of local zebu cows (0.57) [21]. Although factors for the lower CR of Japanese Black cows are still unclear, some studies have indicated that a decrease in reproductive performance of this breed might be due to genetic improvement programs that have heavily focused on meat quantity and meat quality, especially on beef marbling [1–22].

In this study, BCS and parity have a significant effect (p< 0.05) on CR. Significance of parity in Japanese Black cows has also been described [23], but without showing any significant effect of BCS on CR. Furthermore, in the current study, the interaction between BCS and each of season and AI number was significant (p<0.05); however, no significant effect of the interaction between BCS and parity has been shown in the aforementioned study. In contrast, a statitically significant interaction between BCS and parity for CR has been described in Florida beef cattle [24].

Cows with BCS 5 showed the lowest CR in spring and summer. On the other hand, cows with BCS 4 showed the highest CR in summer. Low CR of cows serviced in spring is consistent with the previous studies in terms of seasonal effect on reproductive performace of Japanese Black cows, whereas low CR has been described in cows serviced in spring and winter [19]. Japanese Black cows need more servicings for conception in spring [25].

In terms of AI numbers, CR showed a significant difference between the second and third servicing. As the AI number increased, the CR of cows with BCS 3 and 4 decreased. The lowest CR of cows with BCS 5 was observed in the third AI. This result also agrees with a study on the number of servicings for CR of Japanese Black cattle [19], where a low CR is shown at a second or later insemination. Various factors have been reported for low CR on repeated AI: genetic [26], nutritional [27] and hormonal causality [28,29]. Cows with low BCS that fail to conceive at the first AI need nutritional treatment to improve CR in subsequent AI.

In the present study, search of the literature produced no estimates of heritability of CR in Japanese Black cows. The estimated heritability of CR (0.012 and 0.021) was lower than that reported for Holstein cows (0.027 to 0.049) in Japan [30]. Low DIC of the model with BCS indicated that the model was better than the model without BCS [31]. The statistical significance of BCS for CR comfirms that BCS should be included in the genetic analysis of CR records.

## CONCLUSION

The present study showed that Japanese Black cows with a moderate BCS had a good conception rate. The model with BCS fitted well with the estimation of genetic parameters because of low DIC of heritability that, albeit, was slightly low.

## Figures and Tables

**Table 1:** Summary of records and conception rate

Factor	N	Mean±SEM	Minimum	Maximum
BCS	5,001	3.39±0.01	2	5
Parity	2,114	5.07±0.04	1	18
AI numbers at conception	3,482	1.75±0.02	1	12
Number of cows at farm	114	52.92±5.38	1	322
Conception rate				
All data	6,034	0.52±0.01	0	1
BCS 2	349	0.48±0.03		
BCS 3	2,448	0.52±0.01		
BCS 4	2,079	0.51±0.01		
BCS 5	127	0.47±0.04		

SEM, standard error of the mean; BCS, body condition score.

**Table 2:** Type III tests of significance for factors of conception rate

Factor	Df	Mean square	F value	p-value
BCS	3	0.67	2.81	0.0141
Parity	9	0.43	1.91	0.0472
Season	3	0.58	2.42	0.0636
AI number	3	0.48	2.09	0.1078
BCS×parity	27	0.21	0.94	0.3795
BCS×season	9	0.47	2.03	0.0163
BCS×AI number	9	0.49	2.01	0.0177

BCS, body condition score; AI, artificial insemination.

**Table 3:** Interaction between BCS and season of conception rate (LSM±SE)

Season	BCS			

	2	3	4	5
Spring	0.44±0.05	0.51±0.03^b^	0.48±0.02^b^	0.38±0.07
Summer	0.47±0.06	0.50±0.03^b^	0.56±0.01^a^	0.44±0.08
Autumn	0.51±0.05	0.56±0.01^a^	0.52±0.03^ab^	0.56±0.08
Winter	0.54±0.07	0.53±0.02^ab^	0.50±0.03^ab^	0.51±0.09

BCS, body condition score; LSM, lease squares means; SE, standard error.

a,b Different superscripts within a column show significant differences (p<0.05).

**Table 4:** Interaction between BCS and AI number of conception rate (LSM±SE)

AI number	BCS			

	2	3	4	5
1	0.49±0.04	0.56±0.02^a^	0.55±0.01^a^	0.49±0.06^a,b^
2	0.53±0.05^x^	0.55±0.02^a,x^	0.48±0.02^a,y^	0.55±0.08^a,x^
3	0.45±0.07^x^	0.47±0.03^a,x^	0.44±0.03^b,x^	0.29±0.09^b,y^
≥4	0.36±0.06	0.31±0.03^b^	0.43±0.03^b^	0.50±0.09^a,b^

BCS, body condition score; AI, artificial insemination; LSM, lease squares means; SE, standard error.

a,b Different superscripts within a column shows significant differences (p<0.05).

x,y Different superscripts within a row shows significant differences (p<0.05).

**Table 5:** Genetic parameters, Monte Carlo error, and deviance information criterion of heritability of conception rate estimates under threshold model

Parameters^1)^	BCS was included	BCS was excluded
$\sigma_{a}^{2}$	0.0129	0.0233
$\sigma_{pe}^{2}$	0.0234	0.0960
$\sigma_{e}^{2}$	1.0012	1.0013
h^2^ (PSD)	0.012 (0.018)	0.021 (0.060)
Geweke (p-value)	0.03	0.07
MCE	0.0003	0.0018
DIC	7,896.89	8,112.62

BCS, body condition score; PSD, posterior standard deviation; MCE, Monte Carlo error, DIC, deviance information criterion of heritability.

1) $\sigma_{a}^{2}$, additive genetic variance; $\sigma_{pe}^{2}$, permanent environment variance; $\sigma_{e}^{2}$, residual variance; h^2^, heritability.
